# Sampling Enrichment toward Target Structures Using Hybrid Molecular Dynamics-Monte Carlo Simulations

**DOI:** 10.1371/journal.pone.0156043

**Published:** 2016-05-26

**Authors:** Kecheng Yang, Bartosz Różycki, Fengchao Cui, Ce Shi, Wenduo Chen, Yunqi Li

**Affiliations:** 1 Key Laboratory of Synthetic Rubber & Laboratory of Advanced Power Sources, Changchun Institute of Applied Chemistry (CIAC), Chinese Academy of Sciences, Changchun, 130022, P. R. China; 2 Institute of Physics, Polish Academy of Sciences, Aleja Lotników 32/46, 02–668, Warsaw, Poland; 3 University of Chinese Academy of Sciences, Beijing, 100049, China; University of Queensland, AUSTRALIA

## Abstract

Sampling enrichment toward a target state, an analogue of the improvement of sampling efficiency (SE), is critical in both the refinement of protein structures and the generation of near-native structure ensembles for the exploration of structure-function relationships. We developed a hybrid molecular dynamics (MD)-Monte Carlo (MC) approach to enrich the sampling toward the target structures. In this approach, the higher SE is achieved by perturbing the conventional MD simulations with a MC structure-acceptance judgment, which is based on the coincidence degree of small angle x-ray scattering (SAXS) intensity profiles between the simulation structures and the target structure. We found that the hybrid simulations could significantly improve SE by making the top-ranked models much closer to the target structures both in the secondary and tertiary structures. Specifically, for the 20 mono-residue peptides, when the initial structures had the root-mean-squared deviation (RMSD) from the target structure smaller than 7 Å, the hybrid MD-MC simulations afforded, on average, 0.83 Å and 1.73 Å in RMSD closer to the target than the parallel MD simulations at 310K and 370K, respectively. Meanwhile, the average SE values are also increased by 13.2% and 15.7%. The enrichment of sampling becomes more significant when the target states are gradually detectable in the MD-MC simulations in comparison with the parallel MD simulations, and provide >200% improvement in SE. We also performed a test of the hybrid MD-MC approach in the real protein system, the results showed that the SE for 3 out of 5 real proteins are improved. Overall, this work presents an efficient way of utilizing solution SAXS to improve protein structure prediction and refinement, as well as the generation of near native structures for function annotation.

## Introduction

Biological functions of macromolecules can usually be understood in fine detail on the basis of their atomic structures. Experimental methods including nuclear magnetic resonance (NMR), Cryo-electron microscopy and X-ray crystallography as well as computational algorithms are vigorously developed to provide reliable atomic structures. However, despite the huge progresses that have been made in protein structure prediction and refinement in the last two decades[1,2], fundamental problems associated with simulating and scoring of protein conformations are still far from being properly resolved. In fact, the most recent Critical Assessment of Techniques for Protein Structure Prediction (CASP) experiment indicates that within the last ten years, marginal improvement has been achieved in the predictive accuracy of the overall backbone structure[2] while diverse efforts in protein structure refinement have brought some degree of improvement[3].

Among these efforts, molecular dynamics (MD) simulations equipped with classical force fields are extensively used for studies on mechanisms of protein functions[4,5] and for structure refinement[6,7]. However, MD simulations may lead to unsatisfactory results because of problems in conformational sampling[8], especially when the desired state is energetically unfavorable in the simulation force field[6,9]. Incorporating experimental information, such as NMR short-range atom pair restraints[10,11], Cyro-EM globular contour constraints[12,13] and small angle x-ray scattering (SAXS) shape and size restraints[14,15] into molecular simulations may help overcome this obstacle. In particular, SAXS has been increasingly integrated into molecular simulations owe to its intrinsic merits[16]. SAXS is a robust and manageable technique, which can be applied under near-physiological conditions with no strict limitations on temperature, buffer conditions and macromolecular mass or size[17]. The contemporary computational studies that utilize SAXS experimental data as pseudo-potential functions or scoring functions[18–20] mainly focus on (i) determining the structures of multi-domain proteins and multi-protein complexes on the basis of the atomic structures of their individual subunits[21–23], (ii) improving the accuracy in structure prediction and refinement[14,24,25] and (iii) predicting the conformational ensembles of multi-domain proteins and multi-protein complexes with flexible linkers and loops[15,26–29]. Although these studies greatly facilitate the application of SAXS combined with molecular simulations, it is still not clear how much the SAXS delivered structural information can improve the sampling efficiency in molecular simulations.

In the context of structure-based function annotation, it seems more practical to consider conformational ensembles rather than a static structure determined through crystallography or other techniques. Proteins generally undergo conformational fluctuations on various timescales and amplitudes to perform their biological functions, such as signal transduction, transport and catalysis[30]. It is thus often desired to know a set of representative conformations rather than a single static structure in order to understand how proteins carry out their functions. Furthermore, it has been validated that the success of structure refinement is proportional to the population of native-like decoys[31,32]. Therefore, generating a set of structures with smaller root-mean-square deviation (RMSD) from the target than their initial conformation is of great practical significance. It raises the demand for an universal energy function or a general simulation approach, besides a system-dependent scoring function, to guide simulations toward native structures.

In this work, we develop a hybrid MD-MC simulation approach that incorporates SAXS information to enrich sampling toward a target state. The structural information contained in SAXS-derived data is transformed into a Monte Carlo (MC) pseudo-energy function that acts as a soft restraint in the conformational space search. The use of MD simulations guarantees that the conformational search is limited to energetically achievable states and the simulation structures are physically meaningful. The MC judgment integrated with the MD simulation is used to bias the sampling closer to the target state. We tested our method on 60 structures with 20 identical residues generated by residue substitution to three high-resolution fragments with typical secondary structures (e.g. α-helix, β-sheet and random coil). Under this case, the influence on sampling enrichment aroused from the relationship between target state and the preferable state of a sequence in a given force field that guides the MD simulations can be averaged out. It provides a way to evaluate the power for the integration of SAXS information in the enrichment of sampling toward a target state independent on the force field.

## Methods

**Fig 1:**
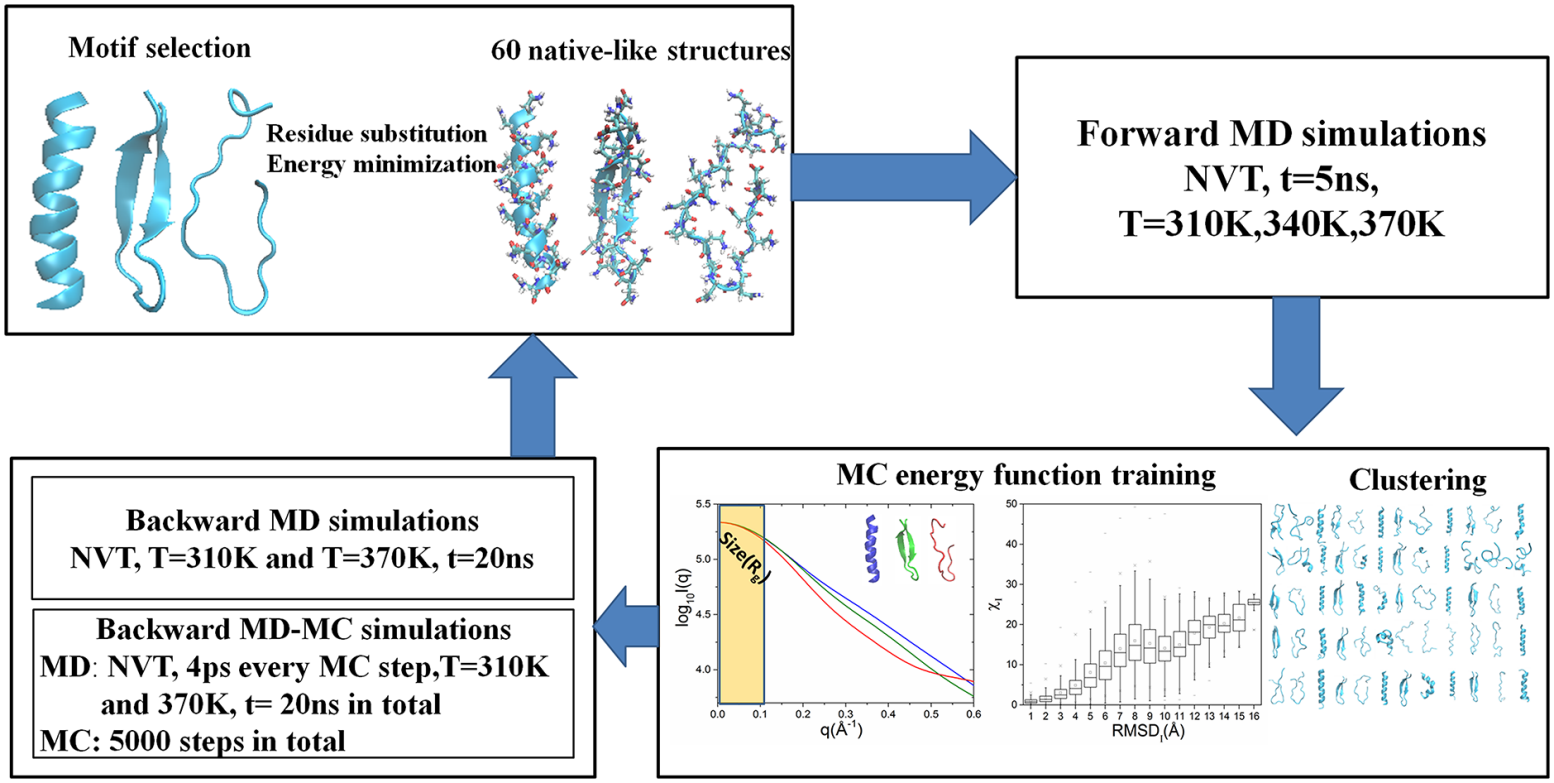
The schematic flowchart of simulations in this work.

Our computational experiments include three parts: (i) Generation of native-like structures. (ii) Forward MD simulations on these native-like structures performed to generate a pool of decoys. Based on these decoys, an optimal SAXS-derived pseudo-potential function for MC simulations is constructed and representative models are selected through clustering. These models are used as the initial structures in the backward simulations. (iii) Backward MD and hybrid MD-MC simulations are performed on the initial structures obtained in (ii). Using the native-like structures generated in (i) as target structures, sampling enrichment of these simulations are evaluated. Here, the terms “forward” and “backward” are used to represent the simulation directions initializing and refining towards the native-like structures, respectively. A flowchart of the experiment is presented in Fig 1, and each of the parts is described below.

### Generation of native-like structures

Native-like structures are selected solely by their secondary structures. We select three structural fragments with typical secondary structures from PDB library: helix (PDB code 1VCS, residues 37–56), sheet (PDB code 1PIN, residues 9–28) and coil (PDB code 1GWP, residues 80–99). The secondary structures are determined using STRIDE[33] [sheet (E, B), helix (H, I, G), coil (C, T)]. The sequences then are substituted by one of the 20 natural amino acids to create mono-residue peptides using Mutator plugin in VMD[34]. These mono-residue peptides have different secondary structural preferences[35], and they may evolve into energetically favorable secondary structure through the MD simulations in a given force field. Therefore, they are good and rational choice to evaluate the overall performance of the hybrid MD-MC method, regardless of whether the target structure is energetically favorable and achievable in MD simulations. Although these mono-residue peptides are not representative for the large diversity of real proteins, the types of residues and secondary structures are comprehensive which make it provide a theoretically sound evaluation. These 60 (3*20) structures are relaxed to get native-like structures through three steps: (i) relocation of atoms and surrounding water molecules with 2000 iterations of a conjugate gradient energy minimization; (ii) equilibration at T = 310K through a 1 ns NVT-ensemble MD simulation; and (iii) equilibration at T = 310K with a 1 ns NPT-ensemble MD simulation. A harmonic potential with the force constant of 1.0 kcal/mol/Å^2^ is applied to all non-hydrogen atoms to minimize the structure change in all the three steps. The final structures are selected as the native-like structures, which are next used as the initial structures in the forward simulations and as the target structures in the backward simulations.

### Forward molecular dynamics simulations

Wide distribution of decoys from the forward simulations is important to select diverse and representative structures for the sampling enrichment study, and to construct a pseudo-energy function that can reliably guide simulations towards the target structure. The forward NVT MD simulations are carried out for 5 ns at 310K, 340K and 370K. In each simulation trajectory, structures are saved every 4 ps, which produce 1250 decoys in each trajectory. Thus, for each mono-residue peptide, a pool with 11250 (1250*3*3) decoys from 3 native-like structures at 3 simulation temperatures is collected.

The initial simulation configuration are prepared using VMD[34] through merging mono-residue peptides into a TIP3P water box[36] with the edge size of 13 Å. Additional sodium or chloride ions are added to neutralize the system. The MD simulations are carried out with the periodic boundary condition using NAMD v2.9[37]. The multiple time stepping integration scheme[38] is used to accelerate electrostatic potential computation, and short-range non-bonded interactions are computed every step using a cutoff of 10 Å with a switch distance of 8 Å. Long-range electrostatic interactions are calculated using the particle-mash Ewald method with a grid spacing of 1 Å^-1^ by every 2 steps. The integration time step is of 2 fs with hydrogen atoms optimization using SHAKE[39,40]. Langevin dynamics for all non-hydrogen atoms is used to keep constant temperature, and the damping coefficient is 1 ps^-1^. The Nose′-Hoover Langevin piston[41] with an interval of 200 fs and a damping timescale of 100 fs are used to maintain a constant pressure at 1 atm.

### Clustering

The clustering of decoys from the forward MD simulations is performed using SPICKER[32] with the initial cut-off RMSD of 4 Å. The cut-off RMSD can be self-adjusted to satisfy the condition that the first and largest cluster (Top 1) cover 15%–70% of all input structures. The final cut-off RMSD is 4 Å for all mono-peptides except for the poly-Gly, which is 4.6 Å. For each mono-residue sequence, the center structures in the three most populated clusters (Top 3 models) are selected as the initial structures for the backward simulations.

### Backward simulations

**Fig 2:**
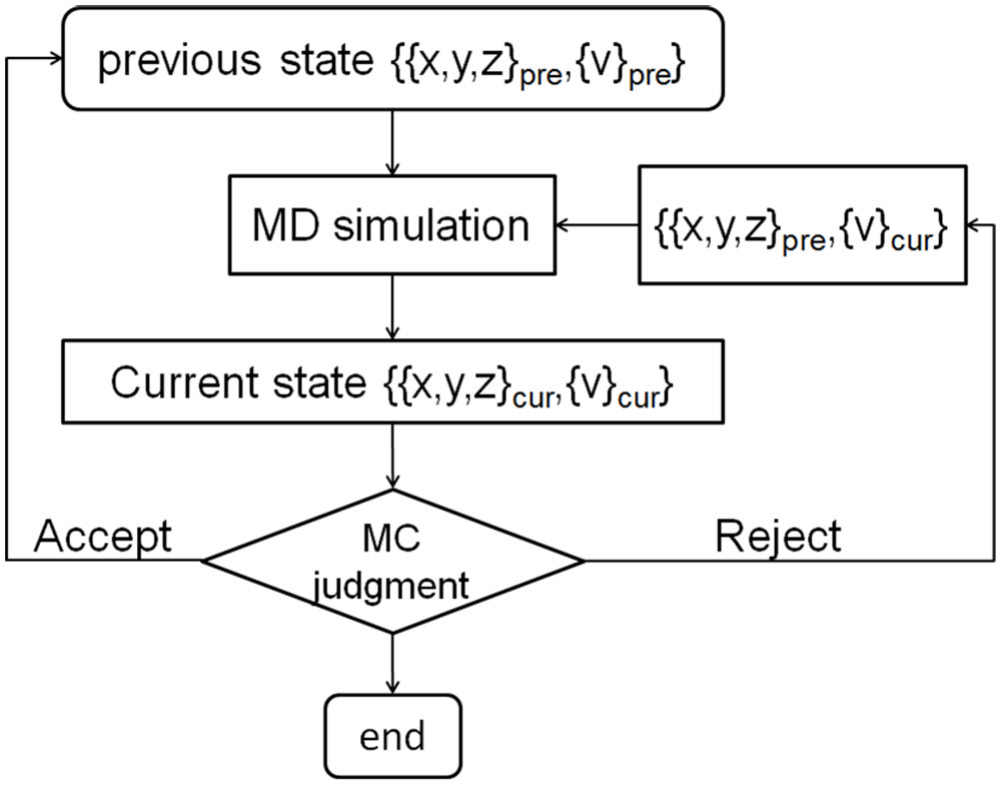
The illustration of MD-MC iterations in the hybrid simulation.

Sampling enrichment is evaluated by comparing the hybrid MD-MC and the parallel MD in the backward simulations, i.e. both the MD-MC simulations and the parallel MD simulations have the same initial structures. The frame of the hybrid MD-MC simulations is presented in Fig 2.

The consecutive MC judgments are made every 4 ps in the MD simulation trajectory, and the decoys are saved after each MC judgment for results analysis. To make a MC judgment, the SAXS intensity profile for a given structure is computed using Fast-SAXS-pro[42]. The acceptance probability in the MC judgment is given by the Metropolis criterion[43], i.e., min{exp[-(E_n_-E_n-1_]/*k*_B_T), 1}. Here, T is the simulation temperature, *k*_B_ is the Boltzmann constant, and E_n_ is the pseudo-energy function for the structure at the n^th^ MC iteration. The pseudo-energy function is taken to be proportional to the discrepancy in the scattering intensity profiles between the target structure and the n^th^ structure in the hybrid simulation.

We perform 20 ns MD and MD-MC simulation at 310 K and 370 K, starting from 60 (20 sequences and Top3) models obtained from the clustering procedure. Since the MD simulations are not biased towards any target structure, we only perform one MD simulation for each of the models (i.e. 60 simulation trajectories). In the case of the MD-MC simulation, where the SAXS-derived information about the target structure is incorporated into the MC pseudo-energy function, we run 60 MD-MC simulations toward three native-like target structures, which resulted in 180 simulation trajectories in total.

Additionally, to provide a solid statistical view on sampling enrichment and minimize the bias from over-sampling at the valley in the energy landscape, we randomly select 600 target structures and 600 initial structures for the MD-MC simulations. These supernumerary simulations are carried out at 370K for 1ns with 0.5ps time interval for the MC judgments in each trajectory.

### Pseudo-energy function

CHARMM22 force field[44] coupled with the CMAP correction[45] is used to guide the MD simulation. It includes geometric terms for the bond lengths, bond angles, dihedral and improper angles, as well as non-bonded terms of Lennard-Jones van der Waals interaction and Debye-Hückel electrostatic potential.

The pseudo-energy function E underlying the MC simulation is taken to be E = ѡ*k*_B_Tχ. Here, the dimensionless parameter ѡ is used to adjust the acceptance ratio in the MC judgment. It is set to 0.2 and 0.4, respectively, for 4ps and 0.5ps intervals in the MD-MC simulations, to keep the MC acceptance ratio of most simulation trajectories above 40% to ensure acceptable simulation efficiency. The discrepancy function χ is a measure of the discrepancy between the SAXS intensity profiles for a given decoy structure and the target structure. It may have alternative forms

$$\chi =\{\begin{array}{llllllll} \frac{c}{N}\overset{q_{\text{max}}}{\underset{q_{\text{min}}}{\sum }}\left|\frac{{\left(q^{n}\text{log}I(q)\right)}_{\text{decoy}}-{\left(q^{n}\text{log}I(q)\right)}_{\text{target}}}{\text{log}I{\left(q_{\text{min}}\right)}_{\text{target}}}\right| & & & & & & & \left(1\right)\\ \text{log}\left[\frac{c}{N}\overset{q_{\text{max}}}{\underset{q_{\text{min}}}{\sum }}\left|\frac{{\left(q^{n}I(q)\right)}_{\text{decoy}}-{\left(q^{n}I(q)\right)}_{\text{target}}}{I{\left(q_{\text{min}}\right)}_{\text{target}}}\right|\right] & & & & & & & \left(2\right) \end{array}$$

Here, *q* denotes the scattering vector, *q*_min_ and *q*_max_ are the boundaries of q which take the values of 0.005 Å^-1^ and 0.6 Å^-1^ respectively, in this work. *N* is the number of the data points in the scattering intensity profiles (*N* = 120), and *c* is an amplification factor for χ (*c* = 1000). I(*q*_min_)_target_ is an approximation of I(*q* = 0)_target_, which is used to normalize the scattering intensity profiles. I(*q*)_decoy_ and I(*q*)_target_ denote the scattering intensity profile for a decoy and the target structure, respectively, and n is an integer number among 0, 1, 2, 3 and 4. The increase of n can gradually accentuate the match of the intensity profiles corresponding to structures at smaller scales, and emphasize the structure information in three classic regions including Guinier[46] (shape and size), Debye[47] (correlation in scattering units) and Porod[48] (interface and surface). Then the pseudo-energy function to guide the MC simulation is selected from one out of the ten formulas as given by Eqs 1 and 2. The choice is made on the basis of the ranking correlation (Spearman coefficient) between χ and RMSD. Here it is worthy to note that the dataset in this work is selected to present general trend though the integration of SAXS intensity profiles into simulation and the energy function to guide MC simulation is expected to have overall correlations and sequence independent.

### Sampling efficiency

Sampling efficiency (SE) for a given simulation trajectory is defined as the probability of finding a simulation decoy with RMSD from the target (R) smaller than RMSD of the initial structure from the target (R_1_). It can be computed as follows

$$SE=\frac{\text{number of decoys with R}<{\text{R}}_{\text{1}}}{\text{number of all decoys}}\;$$ (3)

**Fig 3:**
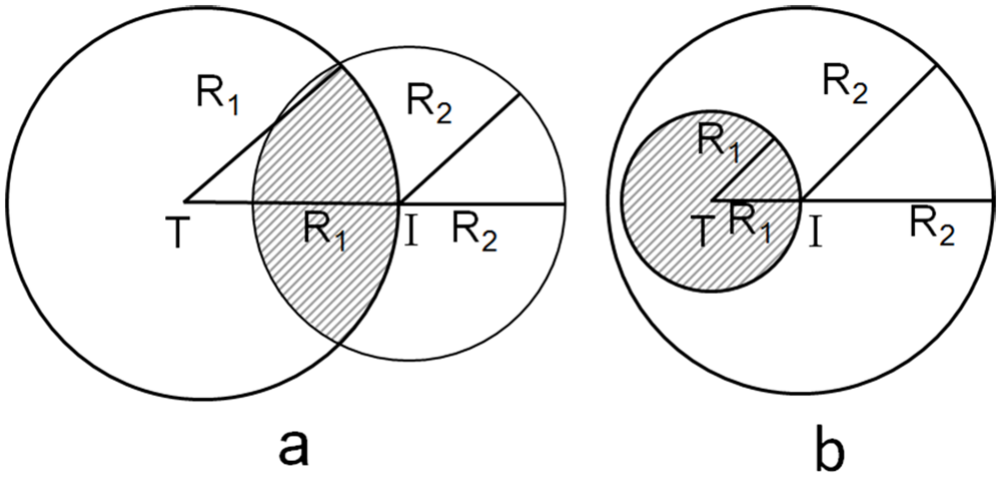
Two-dimensional (2-D) schematic plot for calculating hypothetical sampling efficiency from the initial (I) toward the target (T) structures.

Any simulation trajectory with the known target structure can thus be assigned a specific value of the actual SE. In addition to the actual SE, we also introduce the hypothetical SE, which is based on the assumption of even sampling of objects with spherical symmetry. In a simple geometrical model, the sampling range R_2_ can be represented by the length of a line segment (1D), the radius of a circle (2D) or the radius of a sphere (3D) that encloses 95% of all simulation decoys. R_1_, on the other hand, is given in this simple picture by the distance between the points (in 1D, 2D or 3D) representing the initial and target structures. The hypothetical SE is then given by the ratio of the overlapped length (1D), area (2D) or volume (3D), as illustrated in Fig 3, to the overall sampling length (1D), area (2D) or volume (3D). The hypothetical SE is thus given by the following formulas

$$\begin{array}{ll} SE_{1D}= & \left\{ \begin{array}{ll} \frac{R_{1}}{R_{2}} & \text{ when }R_{2}/R_{1}\geq 2\\ 0.5 & \text{ when }R_{2}/R_{1}<2 \end{array}\right.\\ SE_{2D}= & \left\{ \begin{array}{ll} \frac{R_{1}{}^{2}}{R_{2}{}^{2}} & \text{when }R_{2}/R_{1}\geq 2\\ \frac{1}{\pi }(\frac{R_{1}{}^{2}}{R_{2}{}^{2}}-\frac{1}{2})\theta -\frac{R_{1}}{\pi R_{2}}\sqrt{1-\frac{R_{2}{}^{2}}{4R_{1}{}^{2}}}+0.5\text{ where cos}\theta =1-\frac{R_{2}{}^{2}}{2R_{1}{}^{2}} & \text{when }R_{2}/R_{1}<2 \end{array}\right.\\ SE_{3D}= & \left\{ \begin{array}{ll} \frac{R_{1}{}^{3}}{R_{2}{}^{3}} & \text{ when }R_{2}/R_{1}\geq 2\\ \frac{1}{2}-\frac{3R_{2}}{16R_{1}} & \text{ when }R_{2}/R_{1}<2 \end{array}\right. \end{array}$$ (4)

The hypothetical SE is a continuous function of the ratio R_2_/R_1_. It takes the values between 0 and 0.5, which reflects the assumption about even sampling. The actual SE, on the other hand, takes the values between 0 and 1. Thus, if the actual SE is significantly higher or lower than the hypothetical SE, it indicates uneven or biased sampling.

## Results and Discussion

### Forward simulations

First of all, we attempt to verify the hypothesis of even sampling. Decoys from the forward MD simulations are superposed to their initial structures. The rotational vectors generated in this process are represented by discrete dots in Cartesian coordinate as shown in S1 Fig. The overall sphere-like distribution of these dots supports the assumption that the 180 simulation trajectories in the forward MD simulations of 20 mono-residue sequences with 3 types of secondary structure at 3 temperatures follow even sampling.

We further cluster these decoys according to their sequences and analyze the three most populated clusters (Top3 clusters). The center structures in the Top3 clusters are selected as representative models and termed as Top3 models. The coverage of Top3 clusters (i.e. the fraction of all decoys in the Top3 clusters) and the average of RMSD of each two among the Top3 models are summarized in S1 Table. The representative models cover a broad region in the conformational space with the average of RMSD of any two structures among the Top3 models between 5.3 Å and 11.5 Å. The coverage of the Top3 clusters ranges from 36.24% to 77.79% for all 11,250 decoys generated from 9 simulation trajectories with identical sequence. These results indicate that the decoys generated in the forward MD simulations are broadly distributed. Therefore, those decoys could be used to construct the pseudo-energy function through SAXS intensity profiles, and the Top3 models are suitable to be the initial structures for the backward simulations.

**Fig 4:**
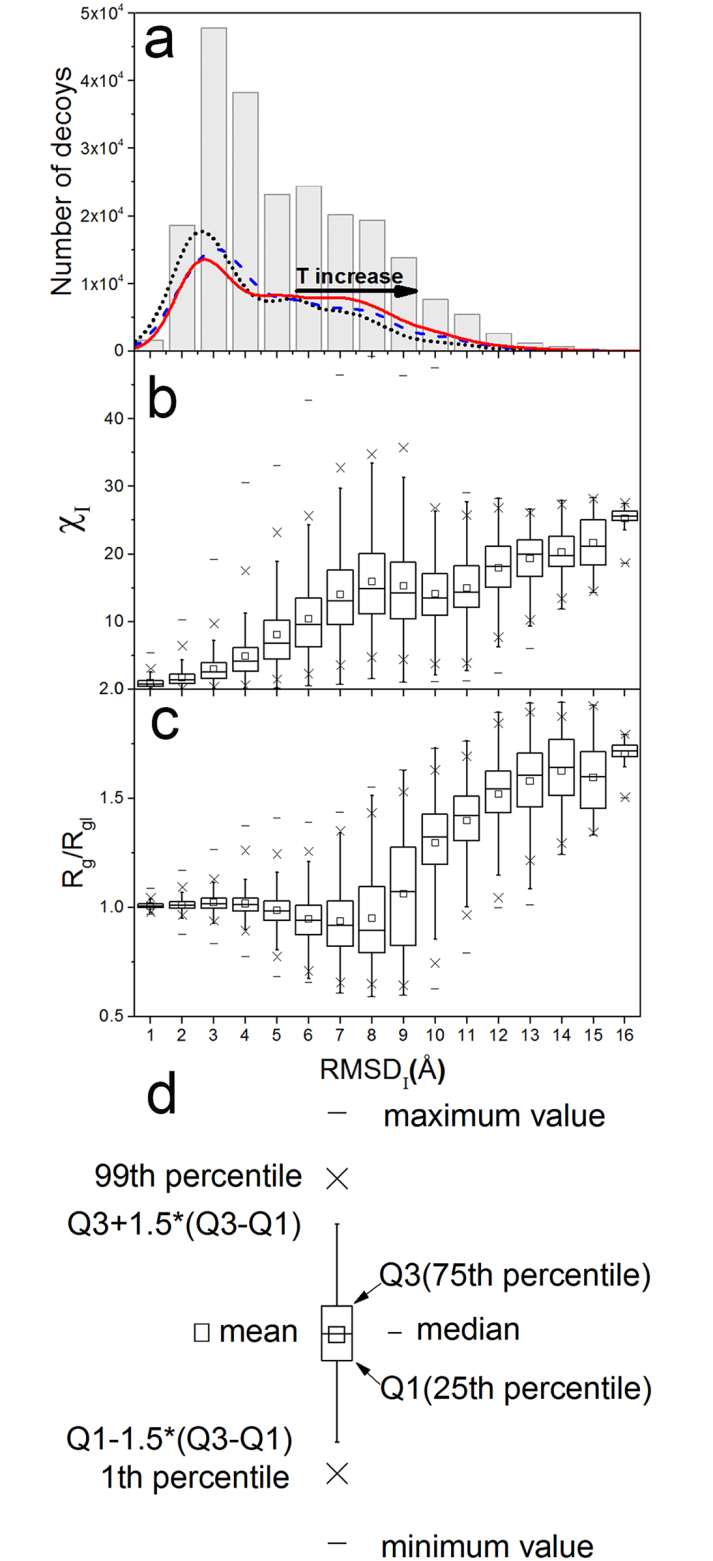
The distribution of decoys, χ_I_ and R_g_/R_gI_ against RMSD_I_. (a) All 22,5000 decoys generated in the forward MD simulations (bars), and decoys in simulations at 310K (dotted lines), 340K (dashed lines) and 370K (solid line); (b) the discrepancy in the scattering intensity profiles (χ_I_); and (c) the size ratio (R_g_/R_gI_). Panel (d) explains the symbols used in panels (b) and (c). RMSD_I_ represents the RMSD of decoys from their initial structures.

To construct the energy function for the hybrid MD-MC simulations, the correlation of χ and RMSD is tested. The RMSD of decoys from their initial structures (RMSD_I_) is calculated and then merged into 1.0 Å bins. The histogram of RMSD_I_ is presented in Fig 4a. It indicates that the majority of decoys departs from their initial structures from 2 to 9 Å in RMSD and significantly enriches in space from 3 to 4 Å. We also observe that the distribution of decoys shift to broader conformation space with elevating temperatures, which is a reasonable deduction from that higher temperature make the energy barrier easier be crossed and so to allow broader sampling space[49].

For each decoy, the discrepancy in scattering intensity profiles (χ_I_) in reference to its initial structure is calculated. The Spearman ranking correlation coefficients between χ_I_ and RMSD_I_ with different definitions of χ_I_ are summarized in Table 1. The results indicate that the correlation decreases with increasing exponent n for both formulas given in Eqs 1 and 2. Eq 1 with n = 0 provides the highest correlation coefficient of 0.819, and so this definition is used later in this study. The distribution of χ_I_ against RMSD_I_ shown in boxplot is presented in Fig 4b and the label of boxplot is depicted in Fig 4d. It can be seen that the correlation between χ_I_ and RMSD_I_ is not monotonous, though they have overall positive correlation. There is a region from 8 to 11 Å, in which they are negatively correlated. This result clearly shows that the implement of SAXS information in simulations will not always improve structure prediction and refinement, which was also observed by other researchers[23]. Additionally, the contribution from large q values is also tested for q_max_ = 0.3 Å^-1^, and the results are summarized in S2 Table. The correlation coefficients for q_max_ = 0.3 Å^-1^ showed the same dependence on the exponent n as that using q_max_ = 0.6 Å^-1^, and the highest correlation coefficient is 0.802. These results indicate the cutoff of large q range has minor impact for the match in structures and scattering intensity profiles.

**Table 1:** Spearman correlation coefficients between the discrepancy functions (χ) and RMSD. The correlations are estimated based on 22,5000 decoys generated in the forward MD simulations. The discrepancy function with ten different forms are for q_max_ = 0.6 Å^-1^.

Exponents (n)	0	1	2	3	4
**Eq 1**	**0.819**	**0.785**	**0.758**	**0.738**	**0.723**
**Eq 2**	**0.800**	**0.768**	**0.719**	**0.678**	**0.651**

Additionally, since the protein size quantified by the radius of gyration (R_g_) is a central parameter that can be steadily obtained from SAXS data, we calculate R_g_ of the decoys from their SAXS intensity profiles. The ratio R_g_/R_gI_ which denotes the relative size change of decoys compared to their initial structures is presented in Fig 4c. The mean of R_g_/R_gI_ has no obvious correlation with RMSD_I_ when it is smaller than 7 Å, and shows a drastic increase when RMSD_I_ > 8 Å. Visual inspection of the simulation structures indicates that this increase normally originates from the peptide unfolding and peptides behaving like random coils. Overall, the radius of gyration of the peptide is less sensitive to changes in RMSD than that of χ, and so we decide to keep χ as the only contributor to the pseudo-energy function. Here, it is worthy to note we found the Pearson correlation coefficient for the R_g_ directly calculated from structures[50] and that using Guinier fitting from SAXS intensity profiles[51] is 0.922. It indicates SAXS profiles can steadily deliver the size information of structures within our dataset.

### Backward simulations

**Fig 5:**
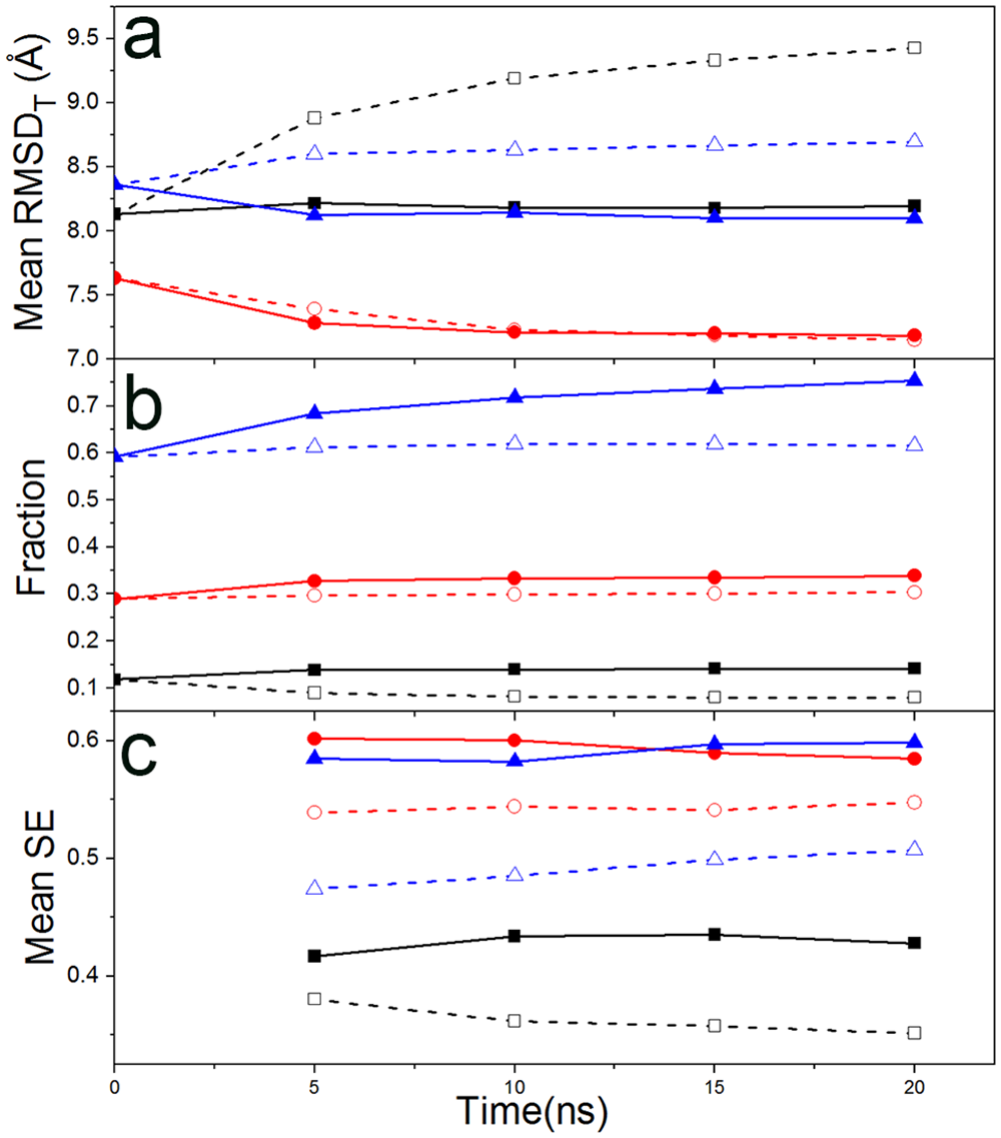
Comparison of RMSD_T_, fractions of secondary structures and sampling efficiency in the backward simulations. Three parameters are calculated from 370K backward simulations against simulation time, for RMSD_T_ (a), fractions of accordant secondary structures to their targets (b) and the actual mean SE (c). The solid symbols are from the hybrid MD-MC simulations, and the empty symbols are those from the parallel MD simulations. The square, circle and triangle present the target with sheet, helix and coil secondary structures, respectively.

The performance of the hybrid MD-MC simulations in sampling enrichment is firstly evaluated. The mean RMSD_T_ and the fractions residues in the secondary structures which are in accord with the target structure, and the mean SE of trajectories in the MD-MC simulations are calculated and compared with those from the parallel MD simulations. These quantities as functions of the simulation time are presented in Fig 5 for simulations at 370K and in S2 Fig for simulations at 310K. Variations of the simulation temperature do not lead to qualitative changes in the overall profiles of these parameters. The mean RMSD_T_ values of decoys from the MD-MC simulations are smaller than those from the MD simulations when the target structures are sheets or coils, while there is no significant difference when the target secondary structure is an alpha helix. For all simulation structures, the reduction in RMSD_T_ are 0.59 and 0.24 Å closer to targets at 370 and 310 K, respectively.

Further, the MD-MC simulations recovered higher fraction of the accordant secondary structure of targets than the parallel MD simulations, as clearly shown in Fig 5b. The improvement is 7.8% and 4.6% at 370 and 310 K, respectively. Although SAXS profiles normally provide marginal information for protein secondary structures, the proper implementation of SAXS still can improve the accuracy of secondary structure modeling. The sampling efficiency of simulation trajectories as a function of simulation time is presented in Fig 5c. The average SE of trajectories in the MD-MC simulations are higher than those in the MD simulations regardless of secondary structures and simulation temperatures. The average SE values for all 180 trajectories in the MD-MC simulations are increased by 6.8% and 1.1% at 370 and 310 K, respectively. The improvement in SE at 370K is statistically significant with a p-value of 10^−3^. Overall, these three parameters prove that incorporation of SAXS profile into the MD-MC hybrid simulations provide superior performance than the parallel MD simulations to enrich simulation decoys towards target structures.

**Fig 6:**
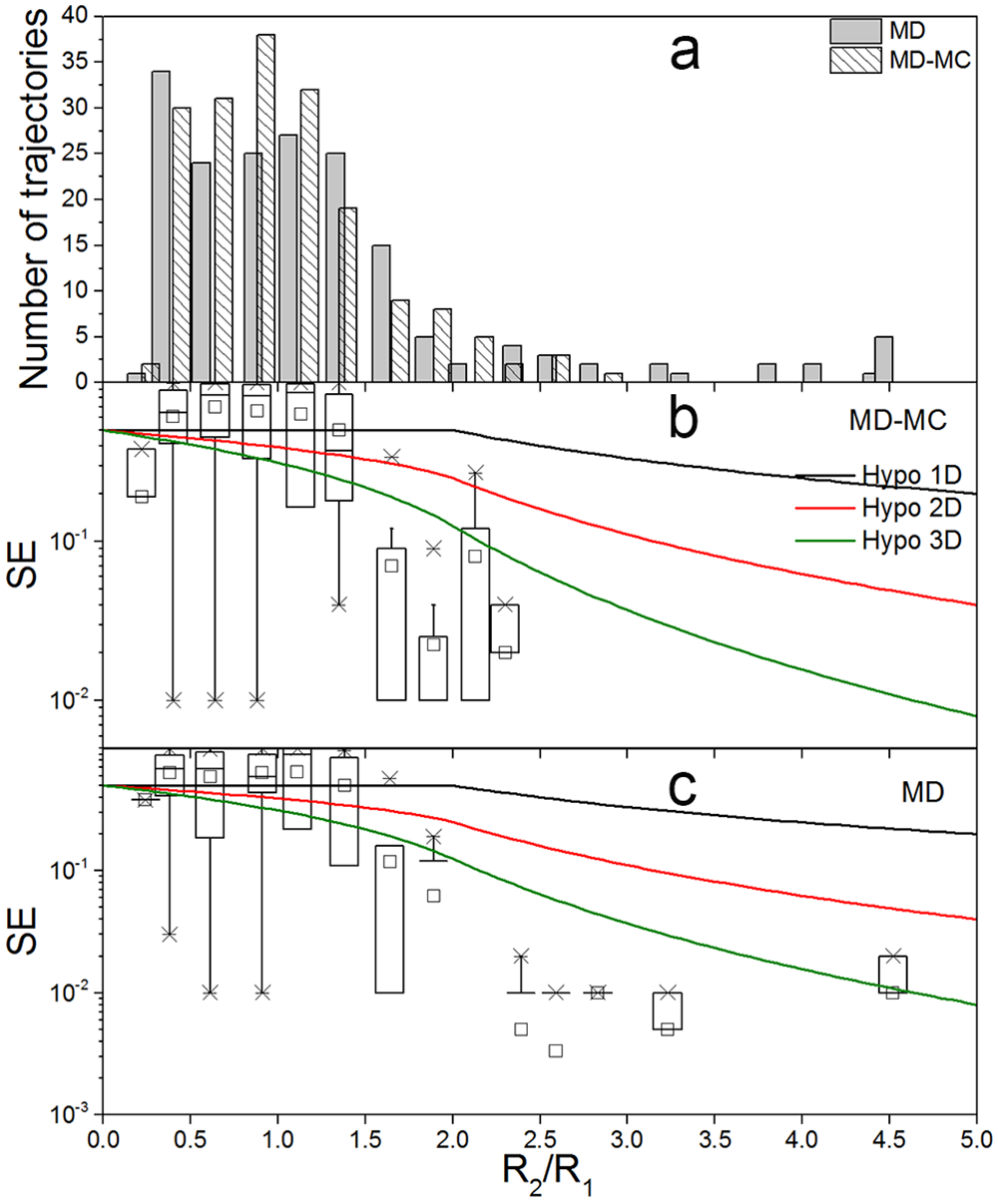
The distribution of trajectories and their SE against R_2_/R_1_. Trajectories from the backward MD-MC simulations and the parallel MD simulations (a); the SE for the MD-MC simulations (b) and the parallel MD simulations (c). The lines present the hypothetical SE curves in 1-D, 2-D and 3-D. The boxplots represent the distribution of the actual SE in each bin.

Fig 4b shows that the relationship between χ and RMSD is not straightforward. For this reason, we group the backward simulation trajectories according to the difference in RMSD between the initial and the target structures (R_1_), and the ratio of sampling range (R_2_) rescaled by R_1_ (R_2_/R_1_). The histogram of R_2_/R_1_ and the actual SE in 180 trajectories of the backward simulations are presented in Fig 6. The corresponding mean values are summarized in Table 2. Since the majority of trajectories are distributed in 0.5 < R_2_/ R_1_ < 2, and the number of trajectories in the group of R_2_/R_1_ > 2 is too small to afford more reliable statistical averages and distributions, the corresponding statistics results of 600 trajectories are also given in S3 Fig and S3 Table. The logarithmic scale is used to highlight the steep decrease in sampling efficiency in the region of R_2_/R_1_>2. The actual SE for both MD-MC and MD simulations decreases with increasing R_2_/R_1_. Most trajectories exhibit the actual SE that has a consistent tendency to the hypothetical SE curves in 3D. The hypothetical SE curve can be regarded as the upper limit for the statistical mean of the actual SE when R_2_/R_1_ >2, because the means of the actual SE are always lower than the hypothetical SE curves. While in the region of R_2_/R_1_ < 2, the actual SE can fluctuate between 0 and 1, which indicate that this region is much affected by the bias of the classic force field. This is also reflected by the value of mean R_2_ which is almost a constant regardless of whether the target can be detected in both MD-MC and MD backward simulations (S3 Table) Overall, with the increase of R_2_/R_1_, where target structures become gradually detectable in simulations, the improvement in SE becomes more prominent by hybrid simulations. When target structures can be entirely sampled in simulation trajectories, i.e., the region of R_2_/R_1_ > 2, the improvement of SE can reach up to 200.0%.

**Table 2:** Sampling efficiency as a function of R_2_/R_1_ and R_1_. The number of trajectories, the mean of R_2_, R_1_ and SE are calculated based on 180 trajectories in the backward MD and MD-MC simulations at 370K. Here, R_2_ is the sampling range in simulations, R_1_ is RMSD between the initial structure and the target structure, SE is the sampling efficiency of a simulation trajectory and the calculated values by Reva’s model are listed in following brackets.

Group	#Trajectories		Mean R_2_(Å)		Mean R_1_(Å)		Mean SE (%)		Improvement (%)
	MD	MD-MC	MD	MD-MC	MD	MD-MC	MD	MD-MC	
R_2_/R_1_(0~1)	84	101	6.08	6.48	10.44	10.37	62.0(69.1)	65.3(68.5)	5.3
R_2_/R_1_(1~2)	72	68	9.10	6.98	7.01	5.51	44.6(30.6)	44.5(16.1)	0
R_2_/R_1_(>2)	24	11	9.07	5.17	2.73	2.30	0.5(0.3)	3.9(0.2)	680.0
R_1_**<7** Å	57	57	7.45	5.37	3.64	3.64	13.2(2.3)	28.9(2.3)	118.9
R_1_**>7** Å	123	123	7.79	7.15	10.08	10.08	62.4(69.7)	65.2(64.1)	4.5
All	180	180	7.68	6.59	8.04	8.04	46.9(44.5)	53.7(44.5)	14.5

**Fig 7:**
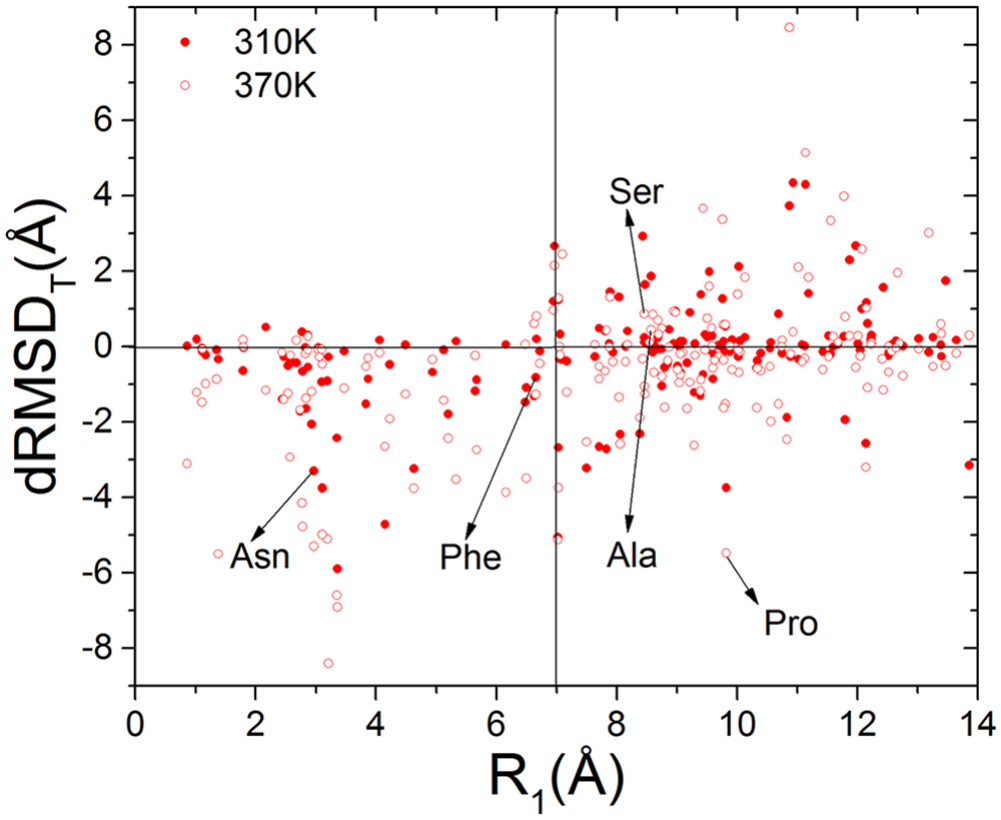
The dRMSD_T_ versus R_1_ between the MD-MC and the MD trajectories with identical initial-target structure pairs. The details of the marked five points are to be presented Fig 8.

In analogy to the classification of easy, medium and hard targets in protein structure prediction and refinement[52], according to the similarity between the initial and target structures (R_1_), the structure similarity of simulation decoys in referent to their targets (RMSD_T_) is computed and analyzed. Two groups are distinguished as easy targets for R_1_ < 7 Å and hard targets for R_1_ > 7 Å, to match the yield point in the χ vs. RMSD curve shown in Fig 4b. The comparison of SE is also listed in Table 2. The improvements due to the hybrid simulations are 118.9% and 4.5% for easy and hard targets, respectively. Besides the overall probability of sampling enrichment, also the reduction of RMSD_T_ for the MD-MC hybrid simulations as compared to the parallel MD simulations, denoted here as dRMSD_T_, as a function of R_1_ is shown in Fig 7. When R_1_ < 7 Å, i.e. for easy targets, 49 and 45 out of 57 cases at 370K and 310K, respectively, have negative dRMSD_T_ values. For hard targets, there are 71 and 55 out of 123 cases at 370K and 310K have negative dRMSD_T_ values. These results indicate higher simulation temperature can help sample in a broad conformational space, and then can improve the sampling enrichment of the hybrid simulations toward target structures. The probability to bring the initial structures to the target structures is higher for easy targets (79%–86%) and relatively lower for hard targets (45%-58%), which agrees with the improvement in SE and the consensus in protein structure prediction and refinement.

Besides, we also compared our actual SE with the calculated values via the theoretical model proposed by Reva et al.[53]. They found that for globular proteins, the probability to generate a random conformation with matched compactness to a target within a given RMSD (R) follows a normal distribution

$$P_{x\leq R}=\frac{1}{\sigma \sqrt{2\pi }}\int_{-\infty }^{R}e^{\frac{-{\left(x-\langle R\rangle \right)}^{2}}{2\sigma^{2}}}dx$$ (5)

Here, <R> and σ, dependent on the size of proteins, are the mean and standard deviation of the distribution of possible RMSD values, respectively. But, as a rational approximation, <R> and σ may be set to 3.333N^1/3^ (N is the number of residues) and 2.0 Å[53,54]. Results in Table 2 clearly show that SE from MD and MD-MC simulations are much better than the random folding according to Reva’s model. It suggests that the hybrid MD-MC method can significantly improve the SE.

**Fig 8:**
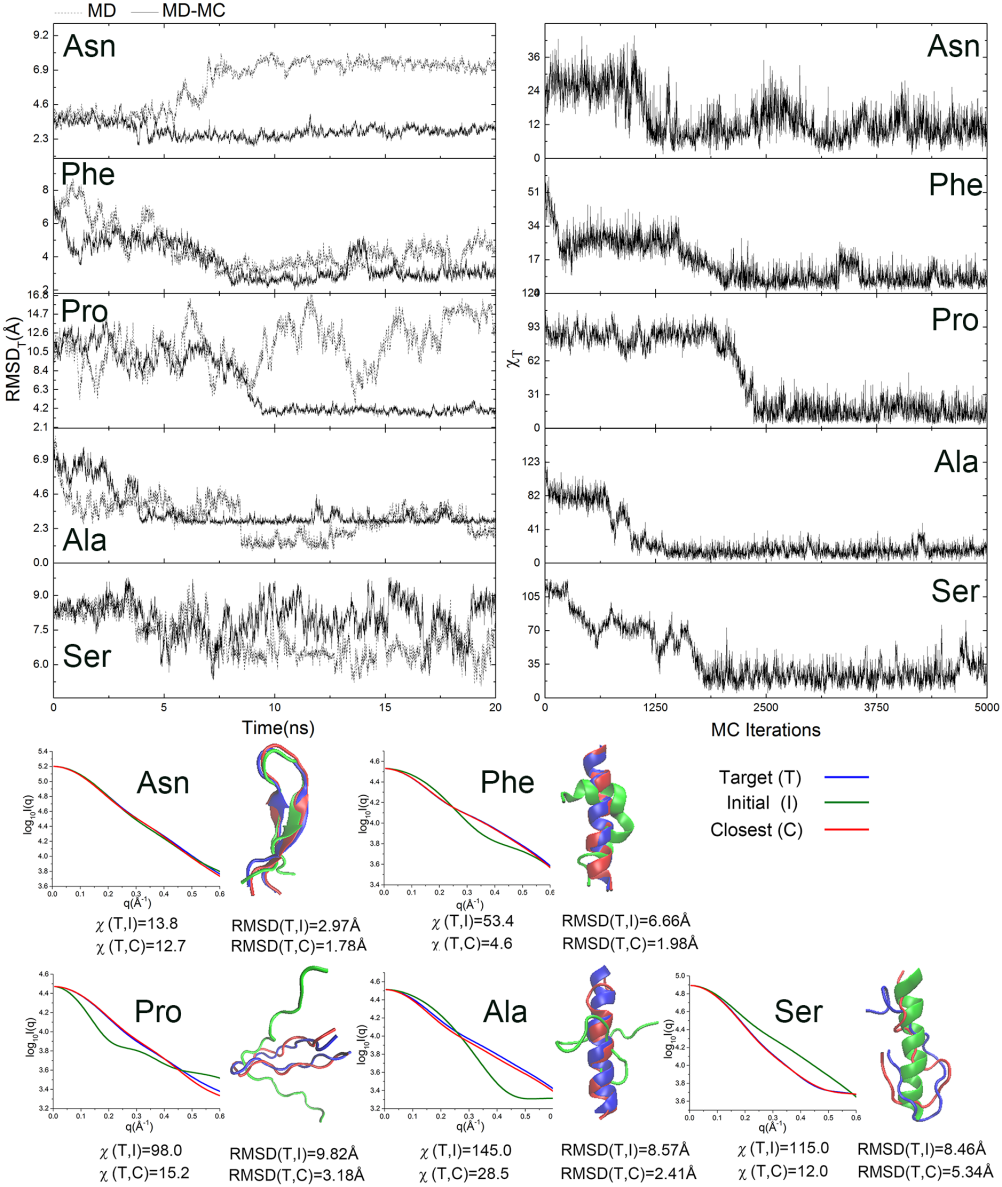
Sampling performance of the MD-MC method in five representative trajectories. The time evolution of RMSD_T_ for MD-MC (solid line) and MD (dash line), the time evolution of χ_T_, as well as 3D structures and SAXS profiles of the initial (I), the target (T) and closest (C) structures for trajectories of Poly-Asn, Poly-Phe, Poly-Pro, Poly-Ala and Poly-Ser are presented.

To demonstrate how did the MD-MC hybrid simulation enrich sampling through the integration of SAXS profiles, we selected five typical simulation trajectories as shown in Fig 8. The associated parameters are listed in Table 3. There are two easy targets (poly-Asn and poly-Phe) and three hard targets (poly-Pro, poly-Ala and poly-Ser), and their locations are marked in Fig 7. For the energy function coupled with the MC simulation, χ_T_ is efficiently minimized and equilibrated within in a relatively short simulation time. The fluctuations of RMSD_T_ are smaller in the MD-MC simulations than that in the parallel MD simulations, which is consistent with the smaller R_2_ in the former simulation reported before. Additionally, the closest simulation decoys to their target structures, the superposition of the two structures and the match in SAXS profiles are also presented. Generally, whether the incorporation of SAXS profiles into simulations can enrich sampling is majorly challenged by the energy landscape given by the MD force field for a particular sequence and the positions of the initial and target structures located in, by the temperature which affects the chances of trapping the simulation trajectories in local energy minima, and by the ratio of R_2_/R_1_ reflecting the probability of the target structures to be sampled.

**Table 3:** The ratio R_2_/R_1_, dRMSD_T_ and dSE for the five representative trajectories shown in Fig 8.

Trajectory	R_2_/R_1_		dRMSD_T_(Å)	dSE
	MD	MD-MC		
Poly-Asn	2.61	1.24	-3.30	0.65
Poly-Phe	1.18	1.11	-0.82	0.03
Poly-Pro	0.73	0.95	-5.48	0.51
Poly-Ala	1.02	1.01	0.44	0.00
Poly-Ser	0.88	0.95	0.86	-0.21

For poly-Asn, since the preferred structure in the MD force field is far from the target structures, the MC perturbations can only suppress the simulations deviate from the target, so the improvement of the hybrid simulation is limited with some extent in the refinement of the sheet secondary structure. While for poly-Phe, both the MD force field and the MC energy function have consistent preferences to the target structure, so the hybrid simulation affords remarkable improvement, especially in the straightening of helix. In both cases, the target structures are partially detectable with R_2_/R_1_ > 1 in both backward simulations. Poly-Pro exhibits large conformational fluctuations in the MD simulations. The hybrid MD-MC simulation provides a globule constraint to stabilize it in a conformation close to the target structure. While poly-Ala has a strong preference to form helix guided by CHARMM force field, both the hybrid MD-MC and the parallel MD simulations reach consistent conformation. Because the target structure is only marginally detectable with R_2_/R_1_ close to 1 in both simulations, the input of SAXS information may disturb the dip of most favorable conformation. The last case is poly-Ser, unlike the above four cases where the MC energy function is dominant or competitive to the MD force field in the hybrid simulation, the MD force field overwhelm the MC judgment perturbation. The results show that the structure of poly-Ser keeps on significant fluctuations during the whole simulations and fails to converge close to the target structure. It raises the awareness of that χ and RMSD are not linearly correlated. Since the spherical average eliminates the one-to-one correspondence between three-dimensional structures and their one-dimensional scattering intensity profiles, degenerate structures and energy states originated from the complex energy landscape for protein folding are still the obstacles for the MD-MC hybrid simulation in providing ensured guidance for variant systems.

### The hybrid MD-MC simulations on real proteins

**Fig 9:**
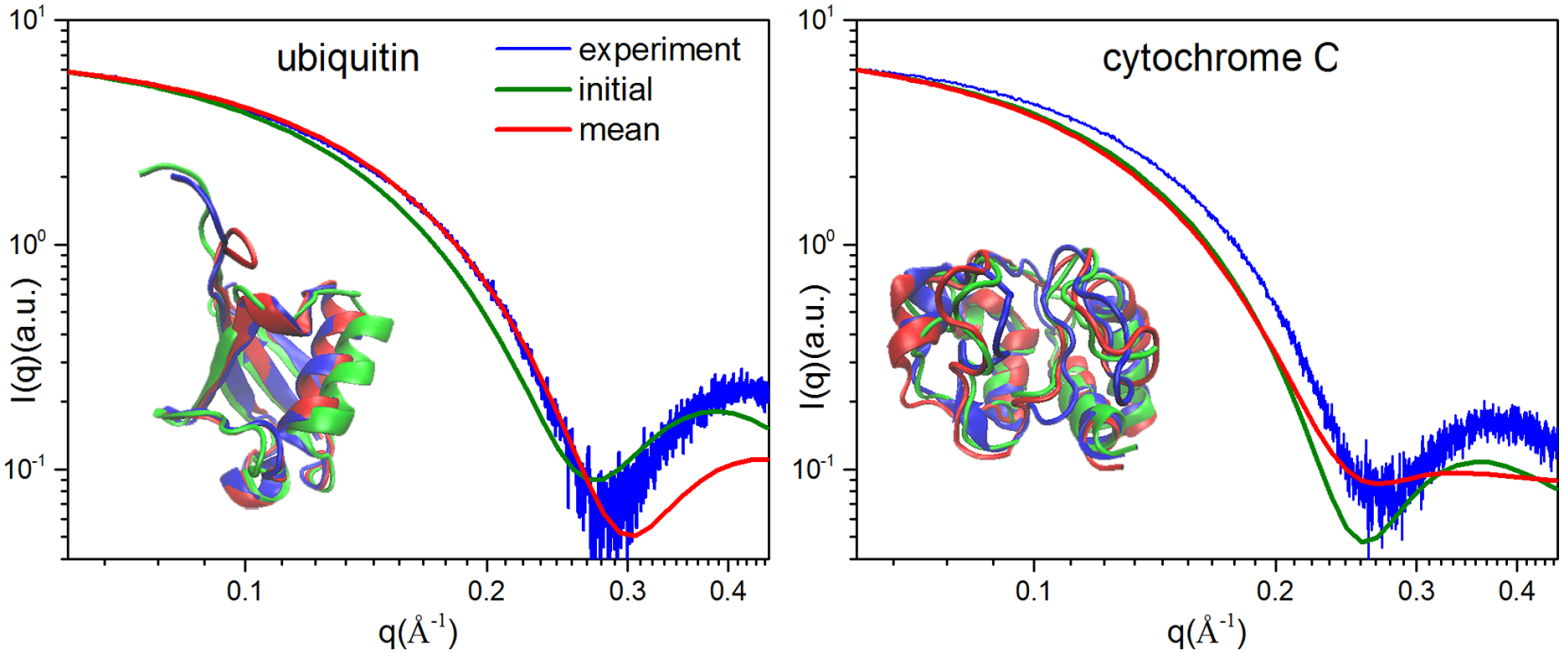
Structural and SAXS profiles comparison among initial (green), target (blue) and simulation (red) structures for ubiquitin and cytochrome C using experimental target SAXS profiles. The calculated SAXS profiles are the average of all 1,250 conformations in the last 5 ns of hybrid MD-MC simulations.

In order to explore the applicability of our method for real protein system, we also carried out the hybrid MD-MC simulations at 370 K for 5 real proteins using calculated SAXS profiles, including bovine antimicrobial peptide (random coil), arenicin-2 (β-sheet), magainin 2 (α-helix), ubiquitin and cytochrome C with multiple secondary structures, whose structures were taken from PDB library as the target structures. The initial structures for three peptides are generated as before. For ubiquitin and cytochrome C, the first model from NMR structures (PDB code: 2LD9 and 1OCD) are regarded as their respective initial structures. Meanwhile, hybrid MD-MC simulations for ubiquitin and cytochrome C also were performed using the experimental SAXS profiles to evaluate the effects of hydration layer and experimental errors, which were download from Small Angle Scattering Biological Data Bank (SASBDB)[55] with the code of SASDAQ2 and SASDAB2, respectively. The simulation results for 5 proteins are listed in Table 4. Comparing to the parallel MD simulations, hybrid MD-MC simulations achieved higher SE and smaller mean RMSD_T_ (denoted by the negative dRMSD_T_) for bovine antimicrobial peptide, magainin 2 and ubiquitin, while there is not obvious improvement for cytochrome C and a decrease for arenicin-2. These are almost consistent with the cases in the 60 mono-residue peptides that the targets of bovine antimicrobial peptide, magainin 2 and ubiquitin belong to easy targets and are detectable due to R_2_/R_1_ > 1, while the target for arenicin-2 belongs to hard target where R_2_/R_1_ < 1. The minimal RMSD_T_ for cytochrome C with 2.98 Å is similar to the result with 3.2 Å achieved by Zheng et al.[14] from the initial structure with R_1_ ~6 Å, in which they adopted a coarse-grained model and kept the secondary structure rigid. We also compared the structures of initial, target and hybrid MD-MC simulations and their SAXS profiles for ubiquitin and cytochrome C, as illustrated in Fig 9. The calculated SAXS profiles are the average of all 1,250 conformations from last 5 ns of hybrid MD-MC simulations. It can be seen that the structures of hybrid MD-MC simulations and target, as well as the calculated and experimental SAXS profiles at q < 0.25 Å^-1^ are almost matched for ubiquitin, while they are not good superposition for cytochrome C. Noteworthily, the good match of average calculated SAXS profiles with the experimental profiles for ubiquitin may indicate that these conformations can represent the ensembles of protein structures, which is necessary for performing their biological function. To further clarify this issue, we averaged the RMSD of each residue of all those conformations, depicted in S4 Fig. It can be found that the C-terminal coiled region, which is the functional region for ubiquitin to perform biological activity[56], is most flexible and undergoes significant conformational fluctuations. Additionally, the Fast-SAXS-pro approach used in this work to calculate SAXS intensity profiles was also compared with CRYSOL[57] to ensure the accuracy. The results presented in S5 Fig validate the accuracy for the SAXS intensity profiles computation method.

**Table 4:** Testing results of the 5 real proteins. R_1_, R_2_/R_1_, dRMSD_T_ and dSE are the mean value calculated based on 3 trajectories in the backward MD and MD-MC simulations for three peptides, while they are from single trajectory for ubiquitin and cytochrome C. The representations of R_1_, R_2_, dSE are presented in Table 2. dRMSD_T_ represents the reduction of RMSD_T_ for hybrid MD-MC simulations as comparing to the parallel MD simulations. The hybrid MD-MC simulations with improvement in either SE or RMSD are bolded.

protein	Target PDB	No. of residues	R_1_ (Å)	R_2_/R_1_		dRMSD_T_ (Å)	dSE
				MD	MD-MC		
bovine antimicrobial peptide	1G89	14	6.43	1.18	1.14	**-0.60**	**0.33**
arenicin-2	2JNI	21	14.01	0.63	0.58	1.35	-0.31
magainin 2	4MGP	23	2.36	1.38	1.19	**-2.98**	**0.68**
Ubiquitinǂ	1UBQ	76	2.77	1.80	1.20	**-2.67**	**0.95**
Ubiquitin*					1.13	**-3.37**	**0.99**
cytochrome Cǂ	1HRC	104	3.15	1.74	1.35	**-0.47**	0.00
cytochrome C*					1.73	0.78	0.00

^ǂ^The SAXS intensity profiles are from direct calculation without the consideration of experimental errors and hydration layer

*The SAXS intensity profiles are from experiments.

To use experimental SAXS profiles, the discrepancy function χ is defined as

$$\chi =\frac{c}{N}\sum_{q_{\text{min}}}^{q_{\text{max}}}\left|\frac{\text{log}I{\left(q\right)}_{\text{cal}}-\text{log}I{\left(q\right)}_{\text{exp}}-\Delta_{\text{offset}}}{\delta I_{\text{log}}(q)}\right|\;$$ (6)

The new terms δI_log_(q) and Δ_offset_ are the experimental errors and the offset between logI_cal_(q) and logI_exp_(q) at q = 0, respectively. In calculated SAXS profiles using the Fast-SAXS-pro, the contributions from hydration layer are considered by water molecules within a shell along protein surface with a thickness of 6 Å (about the sum of 3 Å thick of first hydration layer and 2.8 Å diameter of water) from all non-hydrogen atoms in the protein. The weighting factor w of 4% is used to account the contribution of the hydration layer. The simulation results are shown in Table 4. For ubiquitin and cytochrome C, the SAXS intensity profiles either from experiments or from direct calculation without the consideration of experimental errors and hydration layer are not distinguishable. It suggests that the discrepancy in SAXS intensity profiles is majorly contributed from the change in protein structures.

At last, it is necessary to note that the selection of dataset in this work is to ensure the comprehensive in the types of residues and secondary structures, rather than a set of representative sequences and structures for real proteins. Therefore, this work only provides a theoretically sound evaluation on the performance for the integration SAXS information through the hybrid MD-MC simulations. A stringent test based on carefully selected dataset using similar protocols requires much more efforts and is still undergoing. Further, the merits of solution SAXS technique to characterize the structure of proteins in simulated fluids make it continuously receive more and more attentions. Advancement in the protocol to implement SAXS information in simulations and the rigorous evaluation of performance is still in demand.

## Conclusions

In this work, we developed a hybrid MD-MC method that utilizes the low-resolution structural information contained in SAXS data for sampling enrichment. The MD-MC simulations, on average, could bring the initial structure closer to the target state than the unbiased MD simulations. A hypothetical curve of sampling efficiency (SE) against sampling range (R_2_/R_1_) is proposed. Simulations of 600 trajectories showed a qualitative agreement between the actual and hypothetical SE against R_2_/R_1_. These results indicated that the chances of peptide structure refinement are not just related to similarity between the initial and the target structures, but also dominated by the sampling range in simulations. We found that the MD-MC method is most effective for easy targets with R_1_ < 7 Å and when the target could be detected in the simulation trajectories. The improvement can have over 79% probability to reduce the RMSD to target structures and reach more than 200% in the enrich of SE. Higher simulation temperature can strengthen the superior of the MD-MC hybrid simulation comparing to the parallel MD simulations. Overall, this work presents a way of utilizing experimentally accessible information on target structures to improve protein structure refinement and function annotation.

## Supporting Information

S1 Fig Sampling distribution in the forward MD simulations. Samplings are described by rotational vectors from all decoys generated from the forward MD simulations. Three axes are in the unit of Å.
(TIF)

S2 Fig Comparison of RMSD_T_, fractions of secondary structures and sampling efficiency in the backward simulations. Three parameters are calculated from 310K backward simulations against simulation time, for RMSD_T_ (a), fractions of accordant secondary structures to their targets (b) and the actual mean SE (c). The solid symbols are from the hybrid MD-MC simulations, and the empty symbols are those from the parallel MD simulations. The square, circle and triangle present the target with sheet, helix and coil secondary structures, respectively.
(TIF)

S3 Fig The distribution of trajectories and their SE against R_2_/R_1_. 600 trajectories from the MD-MC simulations and the parallel MD simulations (a); the SE for the MD-MC simulations (b) and the parallel MD simulations (c). The lines present the hypothetical SE curves in 1-D, 2-D and 3-D. The boxplots represent the distribution of the actual SE in each bin.
(TIF)

S4 Fig The average RMSD of each residue in ubiquitin over 1,250 conformations in the last 5ns MD-MC simulations which are using experimental target SAXS intensity profiles. (TIF)

S5 Fig SAXS profiles comparison between the experimental (black) and calculated profiles by Fast-SAXS-pro (red) and CRYSOL (olive and magenta) for ubiquitin (PDB code: 1UBQ) and cytochrome C (PDB code: 1HRC). w is the weighting factor accounting for the excess electron density of the 6 Å hydration layer.
(TIF)

S1 Table The coverage of the Top3 clusters and the average of RMSD of each two among the Top3 models for different sequences. (DOC)

S2 Table Spearman correlation coefficients between the discrepancy functions (χ) and RMSD. The correlations are estimated based on 22,5000 decoys generated in the forward MD simulations. The discrepancy function with ten different forms are for q_max_ = 0.3 Å^-1^.
(DOC)

S3 Table Sampling efficiency as a function of R_2_/R_1_. The number of trajectories, the mean of R_2_, R_1_ and SE are calculated based on 600 trajectories at 370K. Here, R_2_ is the sampling range in simulations, R_1_ is RMSD between the initial structure and the target structure, SE is the sampling efficiency of a simulation trajectory.
(DOC)
