# Supplementary figures and images for: Sampling Enrichment toward Target Structures Using Hybrid Molecular Dynamics-Monte Carlo Simulations
Source: PLoS One. 2016 May 26;11(5):e0156043. doi: 10.1371/journal.pone.0156043 (PMC4881967; doi:10.1371/journal.pone.0156043)

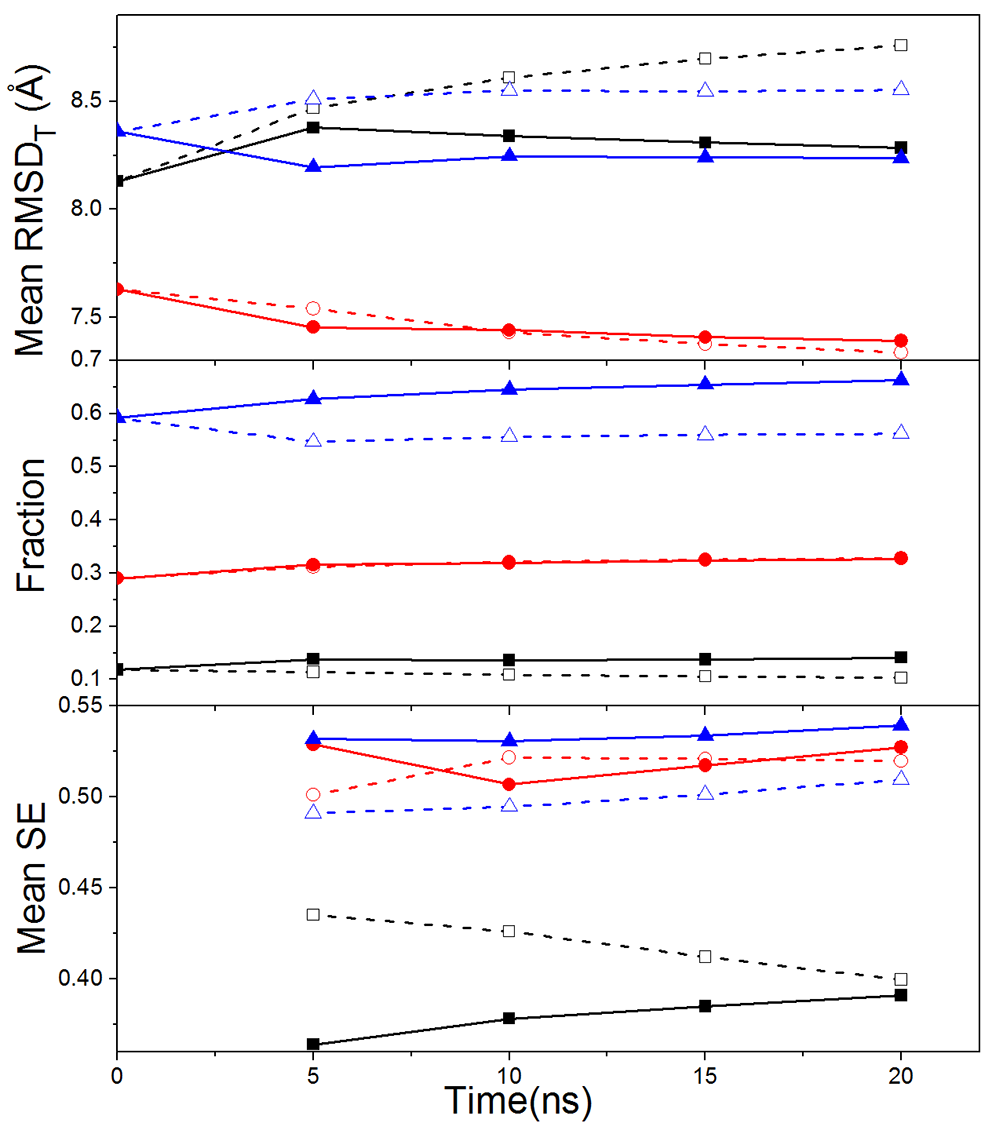

Supplement: S2 Fig — Three parameters are calculated from 310K backward simulations against simulation time, for RMSDT (a), fractions of accordant secondary structures to their targets (b) and the actual mean SE (c). The solid symbols are from the hybrid MD-MC simulations, and the empty symbols are those from the parallel MD simulations. The square, circle and triangle present the target with sheet, helix and coil secondary structures, respectively. (TIF) [file pone.0156043.s002.tif]

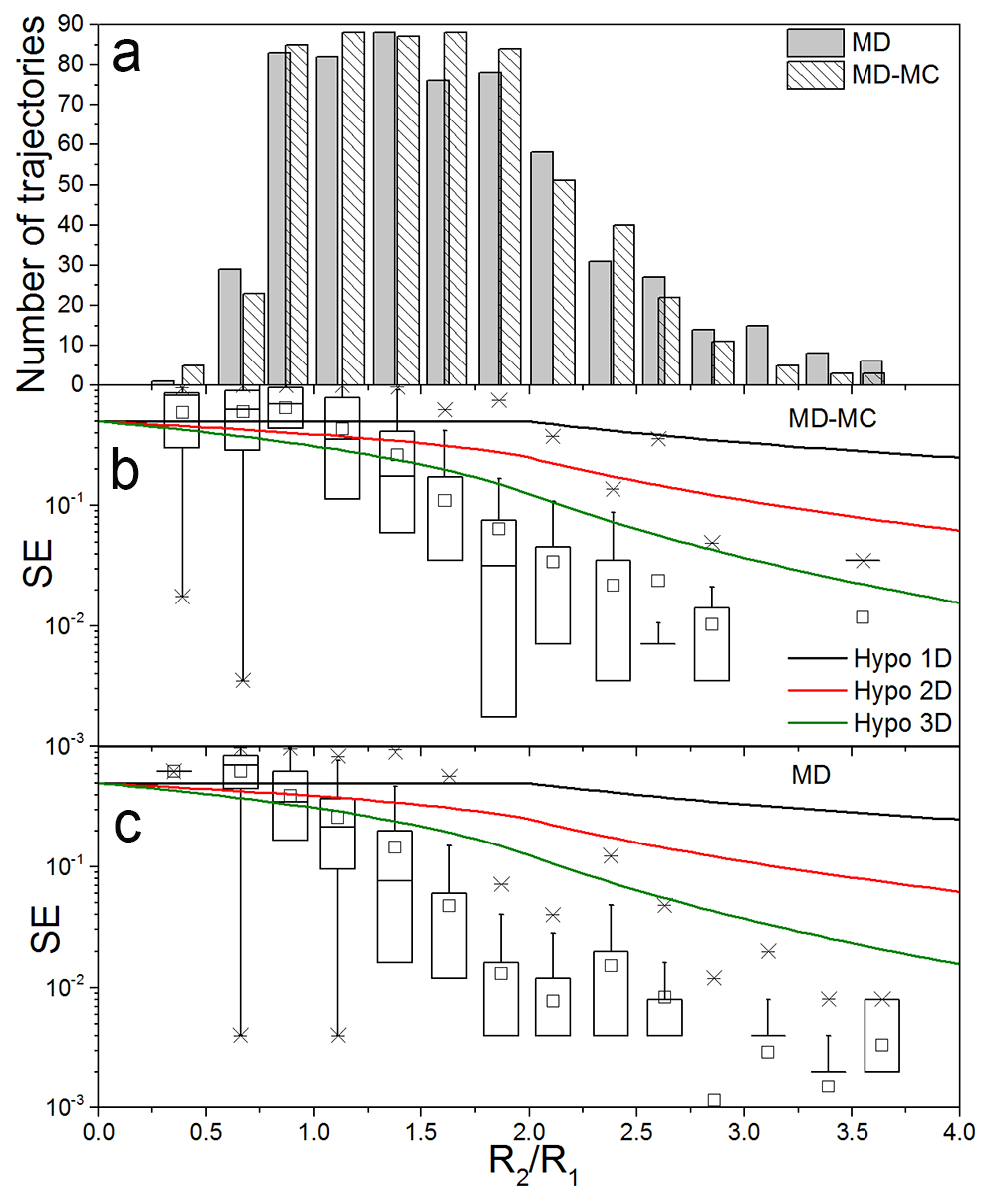

Supplement: S3 Fig — The lines present the hypothetical SE curves in 1-D, 2-D and 3-D. The boxplots represent the distribution of the actual SE in each bin. (TIF) [file pone.0156043.s003.tif]

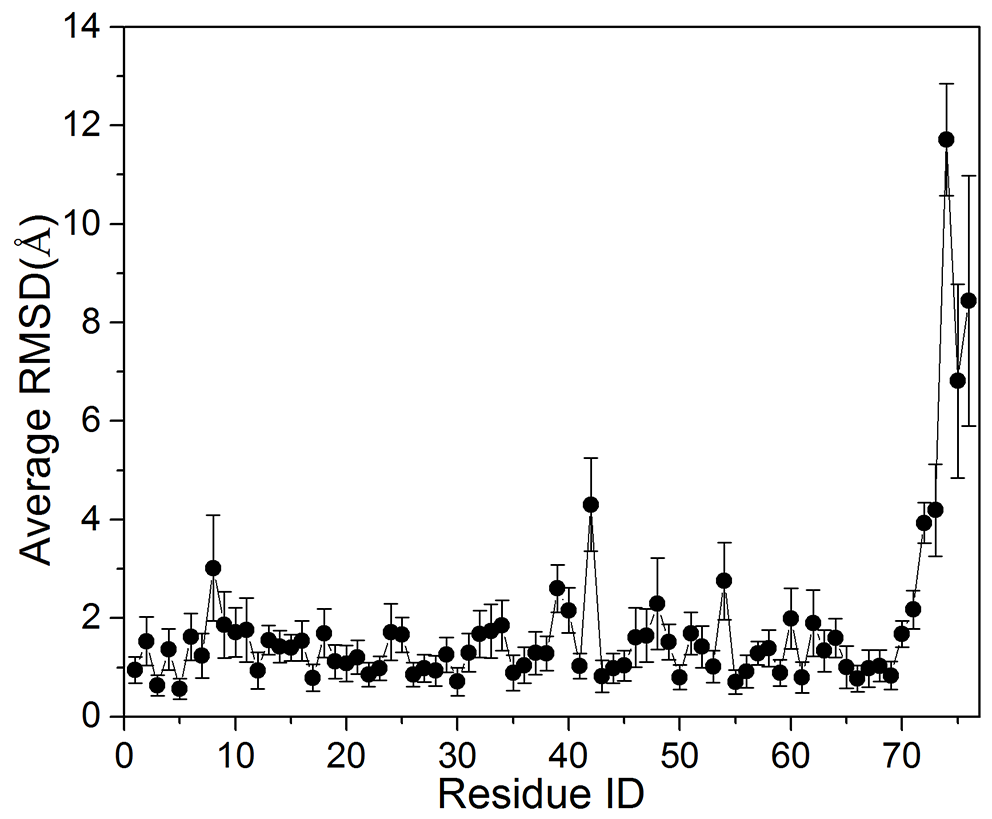

Supplement: S4 Fig — (TIF) [file pone.0156043.s004.tif]

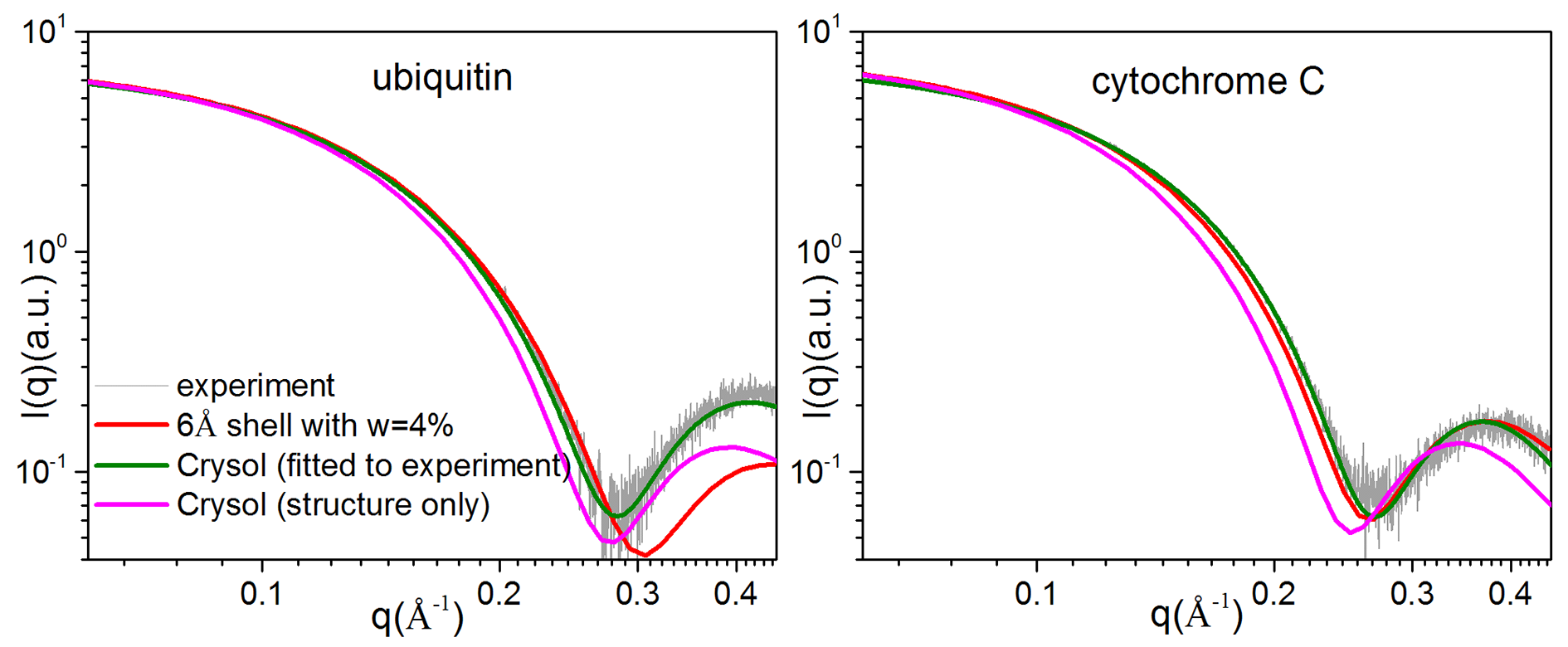

Supplement: S5 Fig — w is the weighting factor accounting for the excess electron density of the 6 Å hydration layer. (TIF) [file pone.0156043.s005.tif]
