# Supplementary material for: Sampling Enrichment toward Target Structures Using Hybrid Molecular Dynamics-Monte Carlo Simulations
Source: PLoS One. 2016 May 26;11(5):e0156043. doi: 10.1371/journal.pone.0156043 (PMC4881967; doi:10.1371/journal.pone.0156043)
Supplement: S1 Table — (DOC) [file pone.0156043.s006.doc]

S1 Table. The coverage of the Top3 clusters and the average of RMSD of each two among the Top3 models for different sequences.

| Residues | Coverage(%) | RMSD(Å) | Residues | Coverage(%) | RMSD(Å) |
| --- | --- | --- | --- | --- | --- |
| ALA | 53.45 | 7.90 | LEU | 72.32 | 10.54 |
| ARG | 70.39 | 11.43 | LYS | 67.15 | 11.34 |
| ASN | 65.29 | 9.65 | MET | 70.19 | 9.81 |
| ASP | 57.71 | 9.76 | PHE | 43.58 | 7.89 |
| CYS | 71.98 | 9.50 | PRO | 41.66 | 6.12 |
| GLN | 69.18 | 10.48 | SER | 52.34 | 9.86 |
| GLU | 77.79 | 10.23 | THR | 48.55 | 9.54 |
| GLY | 36.24 | 5.30 | TRP | 67.94 | 11.48 |
| HSE | 57.59 | 10.65 | TYR | 49.94 | 10.17 |
| ILE | 62.29 | 9.30 | VAL | 74.76 | 11.53 |
