# Supplementary material for: Sampling Enrichment toward Target Structures Using Hybrid Molecular Dynamics-Monte Carlo Simulations
Source: PLoS One. 2016 May 26;11(5):e0156043. doi: 10.1371/journal.pone.0156043 (PMC4881967; doi:10.1371/journal.pone.0156043)
Supplement: S2 Table — The correlations are estimated based on 22,5000 decoys generated in the forward MD simulations. The discrepancy function with ten different forms are for qmax = 0.3 Å-1. (DOC) [file pone.0156043.s007.doc]

**S2 Table.Spearman correlation coefficients between the discrepancy functions (χ) and RMSD.**

| Exponents n | 0 | 1 | 2 | 3 | 4 |
| --- | --- | --- | --- | --- | --- |
| Eq 1 | **0.802** | 0.795 | 0.783 | 0.768 | 0.752 |
| Eq 2 | 0.781 | 0.777 | 0.766 | 0.750 | 0.731 |
