# Supplementary material for: Sampling Enrichment toward Target Structures Using Hybrid Molecular Dynamics-Monte Carlo Simulations
Source: PLoS One. 2016 May 26;11(5):e0156043. doi: 10.1371/journal.pone.0156043 (PMC4881967; doi:10.1371/journal.pone.0156043)
Supplement: S3 Table — The number of trajectories, the mean of R2, R1 and SE are calculated based on 600 trajectories at 370K. Here, R2 is the sampling range in simulations, R1 is RMSD between the initial structure and the target structure, SE is the sampling efficiency of a simulation trajectory. (DOC) [file pone.0156043.s008.doc]

S3 Table. Sampling efficiency as a function of R_2_/R_1_.

| R_2_/R_1_ | #Trajectories | | Mean R_2_(Å) | | Mean R_1_(Å) | | Mean SE (%) | | Improvement (%) |
| --- | --- | --- | --- | --- | --- | --- | --- | --- | --- |
|  | MD | MD-MC | MD | MD-MC | MD | MD-MC | MD | MD-MC |  |
| 0~1 | 113 | 113 | 3.10 | 3.15 | 3.85 | 3.97 | 45.2 | 63.2 | 39.8 |
| 1~2 | 324 | 347 | 3.10 | 3.07 | 2.15 | 2.11 | 10.3 | 21.9 | 126.2 |
| >2 | 163 | 140 | 4.59 | 4.41 | 1.82 | 1.78 | 0.8 | 2.4 | 200.0 |
| All | 600 | 600 | 3.50 | 3.39 | 2.38 | 2.38 | 14.3 | 25.1 | 75.5 |
